# Decreased Efficiency of Neutralizing Antibodies from Previously Infected or Vaccinated Individuals against the B.1.617.2 (Delta) SARS-CoV-2 Variant

**DOI:** 10.1128/spectrum.02706-21

**Published:** 2022-07-05

**Authors:** Chloé Dimeglio, Fabrice Herin, Isabelle Da-Silva, Caroline Gernigon, Marion Porcheron, Sabine Chapuy-Regaud, Jacques Izopet

**Affiliations:** a CHU Toulouse, Hôpital Purpan, Virology Laboratory, Toulouse, France; b INSERM UMR1291 – CNRS UMR5051, Toulouse Institute for Infectious and Inflammatory Diseases (INFINITy), Toulouse, France; c Occupational Diseases Department, Toulouse University Hospital, Toulouse, France; d UMR1295, unité mixte INSERM - Université Toulouse III Paul Sabatier, Centre for Epidemiology and Research in Population Health Unit (CERPOP), Toulouse, France; Pontificia Universidad Católica de Chile

**Keywords:** binding antibodies, neutralizing antibodies, SARS-CoV-2, Delta variant, protection

## Abstract

The neutralizing antibody response is a key component of adaptive immunity and a primary protection against severe acute respiratory syndrome coronavirus 2 (SARS-CoV-2) infection. The increased transmissibility of the SARS-CoV-2 Delta variant and its capacity to cause more severe disease could be linked to a significant reduction in neutralizing antibodies generated during a previous infection or vaccination. We analyzed blood samples from 162 unvaccinated health care workers (HCWs) collected 1 to 3 months postinfection and from 263 vaccinated health care workers 1 month after the last injection. We have compared the neutralizing antibody titers obtained using two virus strains, B.1.160 and B.1.617.2 (Delta variant). Binding antibody concentrations were measured by an immunoassay. The median neutralizing antibody titer against the B.1.160 strain was 128 (interquartile range [IQR], 16 to 256) and 32 (IQR, 8 to 128) against the Delta variant. To obtain a neutralizing antibody titer of 32 or 64, a binding antibody concentration of 182 binding antibody units (BAU)/mL (IQR, 81 to 974) was required with the strain B.1.160, while a concentration of 2,595 BAU/mL (IQR, 1,176 to 5,353) was required with the Delta variant. Our data indicate that antibodies neutralize the SARS-CoV-2 Delta variant 4 times less efficiently than they neutralize an earlier strain. Half of the HCWs had decreased protection from 94% to 76.8% or less for the same total antibody concentration. But neutralization might be correlated with other immune responses. The contributions of other responses, such as those of the T cell and B cell systems, to protection require further investigation.

**IMPORTANCE** Recent studies showed that the neutralizing antibody titer is an important contributor to protection against SARS-CoV-2. With the emergence of new variants, the question arises of maintaining the neutralizing capacities of vaccines and/or of a past infection. We had protective data associated with total antibody concentrations and neutralizing antibody titers for a B.1.160 strain. We showed that to maintain the same levels of protection and, therefore, the same levels of neutralizing antibodies, a total antibody concentration 8.5 times greater is required with the Delta strain. (This study has been registered at ClinicalTrials.gov under registration no. NCT04385108.)

## INTRODUCTION

Several successive severe acute respiratory syndrome coronavirus 2 (SARS-CoV-2) variants have resulted in waves of symptomatic and asymptomatic infections. The Delta variant (lineage B.1.617.2), which emerged in India, was responsible until recently for the majority of infections in many parts of the world. The increased transmissibility of this variant and its capacity to cause more severe disease could be linked to a significant reduction in neutralizing antibodies generated during a previous infection or vaccination (1–4). However, the unvaccinated are still the major drivers of transmission, even in highly vaccinated populations and are themselves at greatest risk of serious disease (5).

The neutralizing antibody (NAb) response is a key component of adaptive immunity and a primary protection against SARS-CoV-2 infection (6–9). The passive transfer of monoclonal (10–14) or polyclonal (15, 16) neutralizing antibodies are also used in therapeutics and prophylaxis. Several *in vitro* assays for these neutralizing antibodies have been developed and used to predict the risk of reinfection or the clinical efficacy of antibody treatment (17). A live-virus neutralization assay is a powerful reference method despite the fact that it is time-consuming and needs a dedicated infrastructure. Our recent study of French health care workers (HCWs) showed that a neutralizing antibody titer greater than 64 gave 94 to 100% protection (18). However, these data were obtained before the spread of the Delta variant in France, and the neutralizing antibody titers were not determined using the B.1.617.2 strain.

We have now compared the titers obtained using the same live-virus neutralization assay and two virus strains, B.1.160 (D614G mutation in the receptor binding domain) and B.1.617.2 (Delta variant). We also analyzed the correlation between neutralizing antibody titers and binding antibody concentrations determined by enzyme-linked immunosorbent assay (ELISA).

## RESULTS

**FIG 1:**
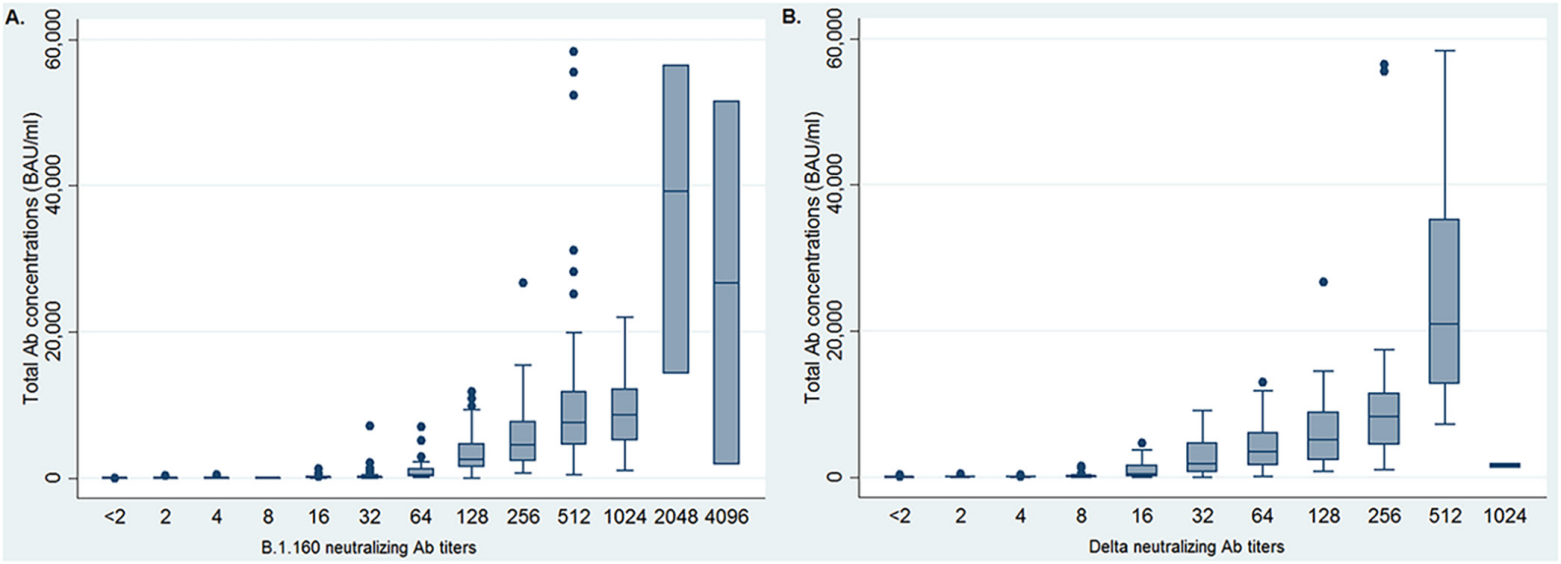
Distribution of total antibody concentrations according to neutralizing antibody titers.

The median total antibody concentration of the 425 HCWs (322/75.8% females; median age, 41 years; range, 19 to 74) using the Wantaï ELISA was 1,027 binding antibody units (BAU)/mL (interquartile range [IQR], 53 to 4,711). Neutralizing antibodies against the B.1.160 strain were detected in 97.7% of subjects and against the Delta variant in 89.9% of subjects. The median neutralizing antibody titer against the B.1.160 strain was 128 (IQR, 16 to 256) (Fig. 1A); it was 32 (IQR, 8 to 128) against the Delta variant (Fig. 1B).

**FIG 2:**
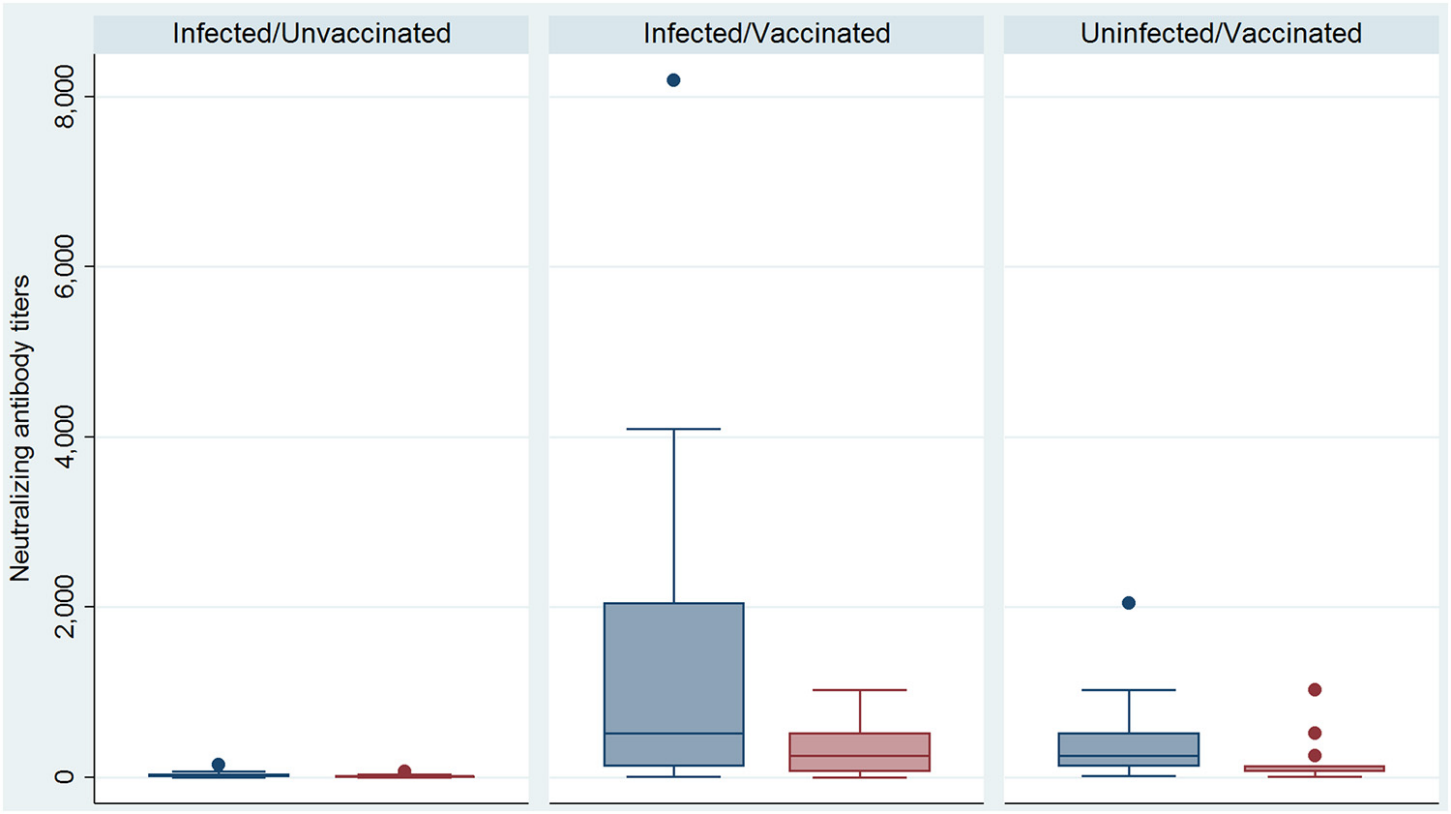
Distributions of neutralizing antibody titers against the Delta variant (red boxplots) and the B.1.160 strain (blue boxplot) according to an individual’s infectious/vaccinated status.

The neutralizing antibody titers of the infected and unvaccinated HCWs for the Delta variant (median, 4; IQR, 2 to 8) were lower than those for the B.1.160 strain (median, 16; IQR, 8 to 32; *P* < 0.01, Wilcoxon signed-rank test) (Fig. 2).

Similarly, the neutralizing antibody titers of infected and vaccinated HCWs against the Delta variant (median, 256; IQR, 64 to 512) were lower than those against the B.1.160 strain (median, 512; IQR, 128 to 2,048; *P* < 0.01, Wilcoxon signed-rank test) (Fig. 2).

The neutralizing antibody titers of vaccinated but not infected HCWs against the Delta variant (median, 128; IQR, 64 to 128) were significantly lower than those targeting the B.1.160 strain (median, 256; IQR, 128 to 512; *P* < 0.01, Wilcoxon signed-rank test) (Fig. 2). The neutralizing antibodies titers against the two strains were not influenced by the type of vaccine given to the HCWs.

**FIG 3:**
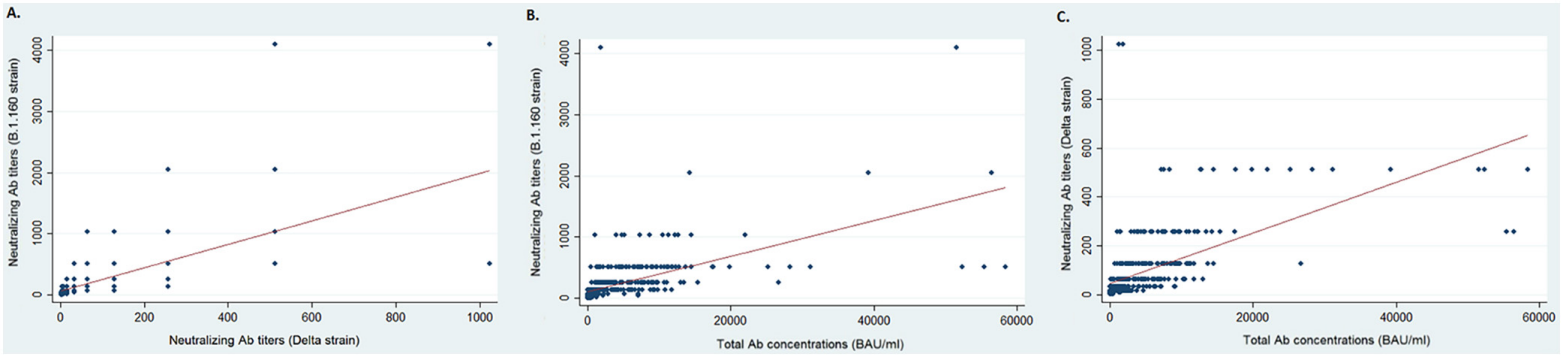
Neutralizing antibody titers and total antibody concentrations. (A) Distributions of neutralizing antibody titers against the B.1.160 strain according to the neutralizing antibody titers against the Delta variant. (B) Distributions of neutralizing antibody titers against the B.1.160 strain according to the total antibody concentrations. (C) Distributions of neutralizing antibody titers against the Delta variant according to the total antibody concentrations.

The Pearson correlation between the titers of neutralizing antibodies against strain B.1.160 and those against the Delta strain was 0.67 (Fig. 3A). The correlation between the concentrations of total binding antibodies and the titers of neutralizing antibodies was 0.56 for strain B.1.160 (Fig. 3B) and 0.59 for the Delta strain (Fig. 3C).

A total antibody concentration greater than or equal to 141 BAU/mL among infected HCWs (vaccinated or not) corresponded to a median neutralizing antibody titer of 64 (IQR, 32 to 96) against strain B.1.160 and to a median titer of 16 (IQR, 8 to 32; *P* < 0.01, Wilcoxon signed-rank test) against the Delta variant. A total antibody concentration greater than or equal to 141 BAU/mL among uninfected/vaccinated HCWs corresponded to a median neutralizing antibody titer of 256 (IQR, 128 to 512) against strain B.1.160 and to a median titer of 128 (IQR, 64 to 128; *P* < 0.01, Wilcoxon signed-rank test) against the Delta variant. Conversely, to obtain a neutralizing antibody titer of 32 or 64 with the strain B.1.160, a binding antibody concentration of 182 BAU/mL (IQR, 81 to 794) was required, while to obtain an NAb titer of 32 or 64 with the Delta variant, a concentration of 2,595 BAU/mL (IQR, 1,176 to 5,353) was required.

## DISCUSSION

These data indicate that an infection with a strain that circulated before the Delta variant and/or a vaccination induce antibodies against the Delta variant with a lower neutralizing capacity, in agreement with previous reports (1, 3). We used blood samples taken 1 to 3 months after infection and 1 month after the last injection to ensure the comparability of the kinetics of the immune response in the case of infection and vaccination. The kinetics of antibody response after a natural infection could be influenced by the abundance of memory B cells increasing between 1 month and 8 months and T follicular helper (TFh) cells (19). Discrepancies between cohort studies could be due to distinct SARS-CoV-2 exposures (9, 20). Correlation between neutralizing antibody titers and total antibody concentrations was not good; this may be due both to the nature of the two variables—one was discrete quantitative (neutralizing antibody titers) and the other continuous quantitative (total antibody concentrations)—and also to the functional character of neutralizing antibodies that accounts for only part of the physical concentration of total antibodies. The difference between the neutralizing capacities of a given concentration of total binding antibodies against strain B.1.160 and the Delta variant was smaller in the vaccinated HCWs than in their infected counterparts (vaccinated or not). Although this result requires confirmation in a larger population, which would make it possible to distinguish the infected from the infected/vaccinated, this suggests that the neutralizing capacity of antibodies induced by isolated spike protein is greater against the Delta variant and, thus, that the Delta variant is less sensitive to serum from naturally infected individuals.

Recent studies suggest that the neutralization titer is an important predictor of vaccine efficacy (6). A recent report analyzing all sequenced strains from symptomatic cases of COVID-19 in England was used to estimate the effect of vaccination on infection (21). The effectiveness of two-dose vaccination against the Delta variant was estimated to be 60% for the AstraZeneca vaccine and 88% for the Pfizer vaccine (21). Our data indicate that antibodies neutralize the SARS-CoV-2 Delta variant from 2 to 4 times less efficiently than they neutralize an earlier strain. Another study found that an NAb titer well below 64 provided 76.8% protection against non-Delta SARS-CoV-2, a titer of 64 to 128 provided 94% protection, and a titer of 256 or more ensured full (100%) protection (18). Thus, half of the uninfected/vaccinated individuals who had NAb titers above 128 after the second injection had only 94% protection against Delta SARS-CoV-2 (18). The protection of the other half of these HCWs decreased from 94% to 76.8% or less for the same total antibody concentration (18). Other neutralization experiments indicate that antibodies elicited by the Pfizer and AstraZeneca vaccines are efficacious against the Delta variant but are about 3-fold to 5-fold less potent than they are against the Alpha variant (22), despite the fact that both vaccines led to the production of similar amounts of antibody. In addition, a recent study on the new Omicron variant (B.1.1.529) showed that the neutralization titers of antibodies to the Omicron variant in serum from plasma donors were 17 to 22 times lower (23). With a 4-fold decrease in neutralizing capacities between strain B.1.160 and the Delta strain, we should have 8.5 times more binding antibodies to reach the same neutralizing capacity of the virus. To be protected against the Omicron strain, we would reach a binding antibody concentration 42.5 times higher.

While previous studies have linked neutralization with protection, neutralizing antibodies are not necessarily totally responsible for protection. Neutralization might be correlated with other immune responses, as suggested by the use of the hemagglutination inhibition titer in influenza (23–25). The contributions of other responses, such as those of the T cell and B cell systems, to protection require further investigation.

## MATERIALS AND METHODS

### Patient serum.

Anonymized blood samples were collected at the Virology laboratory, Toulouse University Hospital before the first Delta variant infections appeared and were biobanked at the hospital Biological Resource Center (certified to NF 596-900 standards). This study was approved by the French Research Ethics Committee Est-111 (COVID BioToul ID-RCB 2020-AO1292-37; ClinicalTrials.gov registration number NCT04385108).

To ensure consistency of the kinetics of the immune response, we used blood samples collected 1 to 3 months postinfection among the nonvaccinated (19) and 1 month after the last injection among the vaccinated HCWs. Blood samples from unvaccinated health care workers (*n* = 162) who had an infection documented by nucleic acid testing of a nasopharyngeal sample (Aptima; Hologic, USA) (26) were collected in July 2020, i.e., 1 to 3 months postinfection in this cohort (27). Samples were collected from vaccinated health care workers (*n* = 263) at the antibody peak following vaccination, i.e., 1 month after the last injection. A total of 217 vaccinated health care workers (HCWs) were seronegative for SARS-CoV-2 prior to vaccination. Of these, 105 (48.4%) were vaccinated with BNT162b2/BNT162b2, 94 (43.3%) with ChAdOx1-S/BNT162b2, and 18 (8.3%) with ChAdOx1-S/ChAdOx1-S. The remaining 46 HCWs were seropositive for SARS-CoV-2 prevaccination and were all given BNT162b2.

### Live-virus neutralization assay.

Neutralizing antibody titers were assessed by endpoint dilution using Vero cells (ATCC CCL-81) and two clinical SARS-CoV-2 strains, B.1.160 and B.1.617.2 (Delta variant). Briefly, 10^4^ Vero cells in 96-well plates were mixed with a virus suspension (100 50% tissue culture infective dose [TCID_50_]) and 2-fold serial dilutions (1:2 to 1:2,048) of the test serum and incubated for 4 days at 37°C. The wells showing a cytopathic effect were identified, and their titers were calculated as the reciprocal of the greatest serum dilution protecting cells from a cytopathic effect.

### SARS-CoV-2 immunoassay.

Total antibodies to SARS-CoV-2 spike protein were assessed using the Wantaï ELISA (Wantaï SARS-CoV-2 Ab ELISA; Beijing Wantaï Biological Pharmacy Enterprise Co., Ltd, Beijing, China) (28–30). ELISA plates were processed in the Bio-Rad EVOLIS system, and a linear relationship was observed between the sample-to-cutoff (S/CO) ratio and antibody concentration for samples in the 1.25 to 14.5 S/CO range. Samples with an S/CO of over 14.5 were diluted in phosphate-buffered saline containing 7.5% bovine serum albumin. Concentrations of binding antibodies are expressed in BAU/mL using the first WHO International Standard for anti-SARS-CoV-2 immunoglobulin (human) as reference for anti-SARS-CoV-2 Ab titers (NIBSC code 20/136; National Institute for Biological Standards and Control, Potters Bar, Hertfordshire, UK) (31, 32).

### Statistical analysis.

Neutralizing antibody titers for strains B.1.160 and Delta were compared by their median and interquartile ranges (IQR) (Wilcoxon signed-rank tests for paired data). Correlations between the two sets of neutralizing antibody titers and total antibody concentrations were evaluated with the Pearson correlation coefficient.

### Data availability.

The strains used were sequenced and the data deposited in GISAID under accession numbers EPI_ISL_804378 (GISAID clade: GH, Pango lineage: B.1.160, Nextclade: 20A.EU2) and EPI_ISL_4276187 (GISAID clade: GK, Pango lineage: B.1.617.2, Nextclade: 21A).
